# Publisher Correction: Local network interaction as a mechanism for wealth inequality

**DOI:** 10.1038/s41467-024-51132-z

**Published:** 2024-08-23

**Authors:** Shao-Tzu Yu, Peng Wang, Chodziwadziwa W. Kabudula, Dickman Gareta, Guy Harling, Brian Houle

**Affiliations:** 1 Office of Population Research, Princeton University, Princeton, NJ USA; 2 School of Demography, The Australian National University, Canberra, ACT Australia; 3 Centre for Transformative Innovation, Swinburne University of Technology, Melbourne, Australia; 4 MRC/Wits Rural Public Health and Health Transitions Research Unit (Agincourt), School of Public Health, Faulty of Health Science, University of the Witwatersrand, Johannesburg, South Africa; 5 Africa Health Research Institute, Durban, South Africa; 6 Institute for Global Health, University College London, London, UK; 7 School of Nursing & Public Health, College of Health Sciences, University of KwaZulu-Natal, Durban, South Africa; 8 CU Population Center, Institute of Behavioral Science, University of Colorado at Boulder, Boulder, CO USA

**Keywords:** Interdisciplinary studies, Economics, Sociology, Developing world

Correction to: *Nature Communications* 10.1038/s41467-024-49607-0, published online 22 June 2024

The system had replaced all the triangles with “!” in the legend in Fig. 4 of this article, and for Fig. 5, the orange square boxes should have been illustrated in red instead; the original and corrected figures are shown below.

The correct version of Fig 4 is:
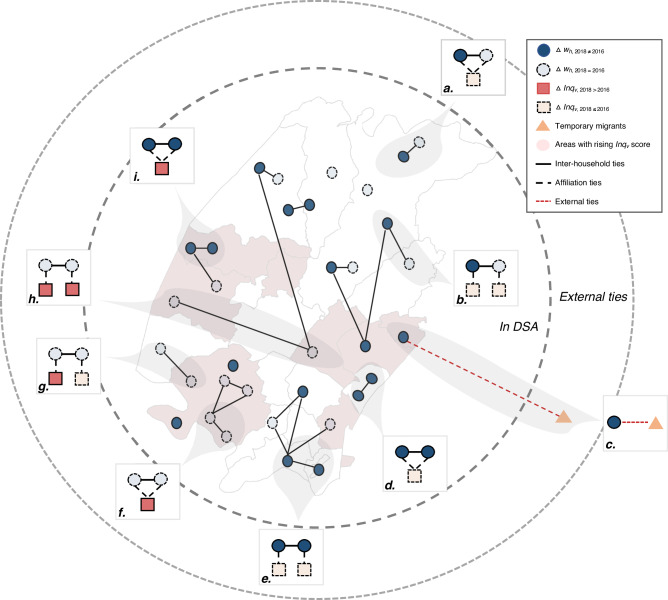


Which replaces the previous incorrect version:


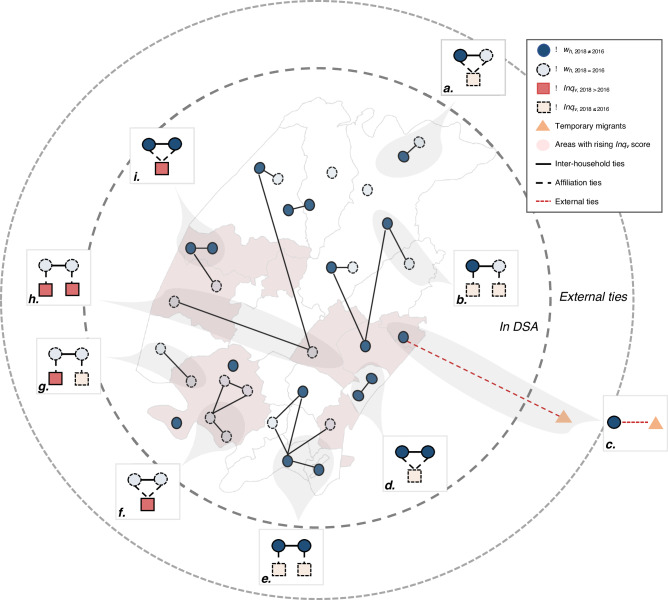


The correct version of Fig 5 is:
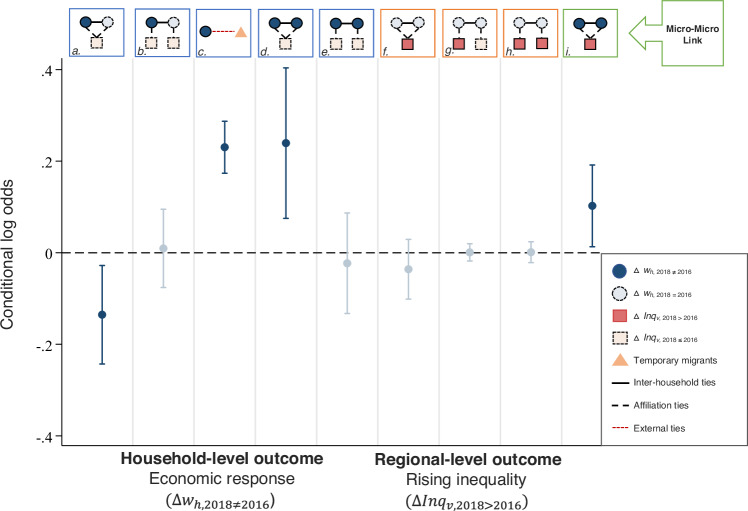


Which replaces the previous incorrect version:
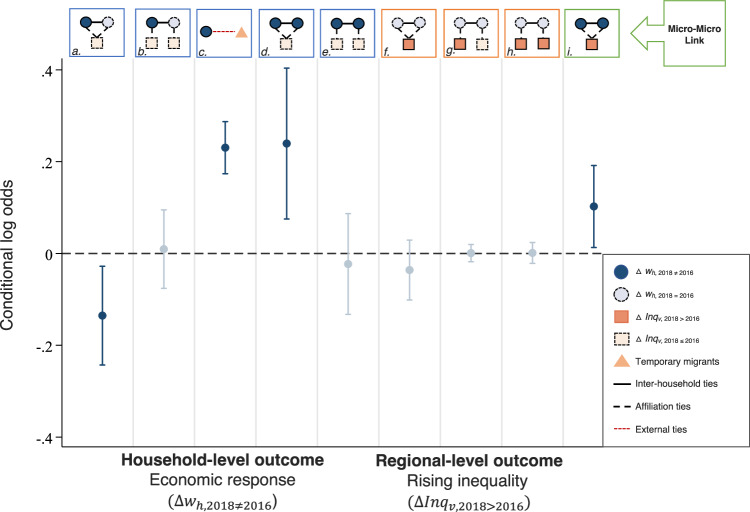


The original article has been updated.

