# Supplementary material for: Local Network Interaction as a Mechanism for Wealth Inequality
Source: Nat Commun. 2024 Jun 22;15:5322. doi: 10.1038/s41467-024-49607-0 (PMC11193797; doi:10.1038/s41467-024-49607-0)
Supplement: Supplementary file 1 — Supplementary Information [file 41467_2024_49607_MOESM1_ESM.pdf]

Supplementary Note 1. Data

Household, homestead, and bounded structures

Different from other Demographic Surveillance and Community Surveys, AHRI DSA establishes three main subjects to enable a better follow-up of individuals in this highly mobile population, as well as to contextualise the complexity and dynamics of rural living arrangements.

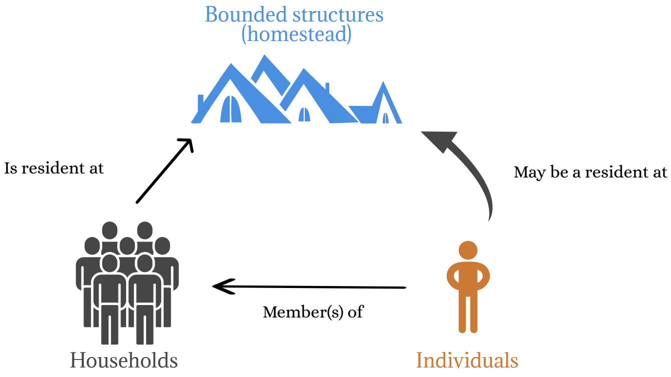

Bounded structures are the most trackable, primary subject of interest in the DSA data conceptualisation. It indicates a physical space of residency (homestead) or facility (school or shop) that links households. Each bounded structure is represented by a point in a geographical information system (GIS) that can be visited routinely and assigned to an administrative unit.

A household is considered as a ‘social group’ that is linked to a bounded structure. AHRI fieldworkers periodically visit all bounded structures to collect data on previously and newly identified households. Respondents (usually the household head or the oldest member) are asked to list all members who belong to the household regardless of their residency status (“*who belong to this group?*”). Households typically comprise a single leader (headship), multiple members with shared responsibilities (care receiving and giving, e.g., childcare), and

a pooling of resources such as food and land (Spiegel 1986; Spiegel et al. 1996; Hosegood, Benzler and Solarsh 2005; Hosegood and Timaeus 2006). Household members are thus usually, but not always, related.

The aforementioned data conceptualisation reflects the 'stretched household' arrangements documented in prior anthropological studies (Spiegel 1986; Spiegel et al. 1996; Russell 2003; Hosegood, Benzler and Solarsh 2005), whereby families work together socio-economically while living apart. In a common case where individuals temporarily migrated to seek formal employment outside of the DSA, they will still be included in the census if they are part of a household (i.e., non-residents or circular labour migrants). Individuals can therefore have multiple, overlapping memberships if they are nominated by multiple households as a member of their social groups.

Network construction and demographics

We utilised the above data conceptualisation to construct the interhousehold social network, using a prospectively collected household membership data from 2000 to 2016. We first organised the household membership data into a two-mode, bipartite matrix (individual-household). We then projected the bipartite matrix to one-mode matrices (household-household) using the Cross-Product Method that reflects a tie between two households if they have nominated the same member.

To better understand the nature of these inter-household ties, we ran a multinomial logistic regression predicting the relative risks of individuals having $multiple$ (1, 2, 3-plus) concurrent household memberships at the baseline year, 2016 ($j = 1, \ldots, \mathcal{J} - 1$). Our choice to use multinomial logistic regression was guided by the proportional odds assumption. The simplification to an ordered logit model was not suitable for our data, as indicated by the Brant test results (p = 0.0015) and further supported by the likelihood ratio test comparing

multinomial and ordered logit models (p = 0.0016). We then added a pair of two-way interactions between respondents' sex, age and its square term. We also included $marital$ status to predict the outcome variable. The estimate can be formally written as

$$log\left(\frac{P(Y=j)}{P(Y=J)}\right) = \alpha + \beta_1 sex + \beta_2 age + \beta_3 age^2 + \beta_4 marital + \beta_5 \, (sex \times age) + \beta_6 \, (sex \times age^2) + \epsilon_j \qquad (2)$$

Results suggest that women are more likely to have multiple memberships at younger ages relative to men; and men are more likely to have multiple memberships at older ages relative to women (Supplementary Figure. 1). Marital arrangements may be one of the reasons that explained this gendered pattern on multiple household memberships at younger ages, as brides' families may still consider them as a member of their household until the groom pays *lobola* (bride wealth) for formal marriage. This in part explains why those in an informal union are marginally more likely to have multiple household memberships relative to the never-married (Supplementary Figure. 1, Panel d). Having concurrent memberships at older ages indicate multiple households nominated a common leader, which may partly be attributable to the practice of polygamy where a husband has multiple wives residing in different homestead (Hosegood, Benzler and Solarsh 2005).

[Supplementary Table 1]

[Supplementary Figure 1]

We further compared this network data with the properties of other theoretical network models, including the random (Erdös and Rényi 1959), scale-free (Barabási and Albert 1999), and small-world models (Watts and Strogatz 1998). We benchmarked the degree distribution, average local clustering coefficient and path length of our observed networks against these

models (Supplementary Table 2 and Supplementary Figure. 2). We began by assembling an Erdős–Rényi random graph, configuring the number of nodes and edge probability to mirror our observed network. Second, we constructed a scale-free network that follows a power-law degree distribution using the Barabási–Albert model. This model maintains the same node count as our observed network and sets the average degree. Finally, a small-world network was constructed, simulating a one-dimensional lattice structure of the observed network size: each node was connected to its nearest two neighbours, with a random rewiring probability of 0.1 to introduce small-world properties such as clustering and short average path lengths, without allowing self-loops or multiple connections between the same nodes.

Unlike a random graph, which in large part features a uniform distribution of connections and low clustering, the observed network exhibits a higher degree and clustering coefficient (Supplementary Table 2). In contrast to a scale-free network, the observed network shares the long-tailed degree distribution but with a lower average degree and higher clustering. This inter-household social network may be more closely aligned with structures observed in the small-world model, yet its higher degree distribution may imply a more hub-like arrangement than a small-world network. To further illustrate varying structural features of the inter-household social network, we visually present the substructure of this network in Fig. 1c of the main text.

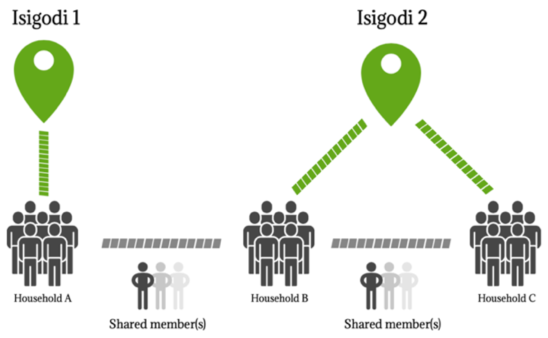

Finally, we linked these households to one of the 23 traditional administrative units, called *Izigodi*. Each *Isigodi* represents a geographic area that is managed by a traditional leader

for their local affairs. An *Isigodi* comprises multiple bounded structures which may also comprise multiple households. *Izigodi* vary in topography, population size, and density of settlement. They are predominantly rural, but also consist of an urban township with a better access to medical facilities, schools, and with better public infrastructure.

[Supplementary Figure 2]

[Supplementary Table 2]

The above data construction resulted in a bipartite, multilevel social network structure with inter-household ties that may exist in the same *isigodi*, or across different *izigodi*. Overall, our inter-household network data consists of 11,834 households and follows a long-tailed degree distribution, with an average degree of proximately 1.72 (SD. = 1.8) per household (see Fig. 1b in the main text). It also consists of 11,834 cross-level, social-geographical ties, linking a household to an *Isigodi*. *Izigodi* vary in size, ranging from about 41 to 1,926 observed households in 2016 and 2018 (Supplementary Table 3).

[Supplementary Table 3]

Supplementary Note 2. Measures

Inequality index: Gini coefficient

We operationalised the Gini index to quantify the levels of wealth inequality for each region. This widely applied inequality index can be formally written as follows (Equation 3):

$$Inq_{v,t} = \frac{\sum_{a=1}^{n}\sum_{b=1}^{n}|w_{h,a} - w_{h,b}|}{2n\sum_{a=1}^{n} w_{h,b}} \quad (3)$$

where v refers to an *Isigodi*, and we denote t as the observed year (2016 and 2018). $w_{h,a}$ and $w_{h,b}$ are the sum of total household asset wealth (w_h) in quintile a and b, with n being the total asset quintiles within an *Isigodi.* The coefficient ranges from 0 (or 0%) to 1 (or 100%), with higher coefficient representing a higher level of disparity in household asset wealth.

Nodal attributes

We adjusted for seven baseline nodal attributes in all analytical models. At the household-level, we adjusted for household $head$ sex (female/others), household baseline $assets$ status (in quintiles), mortality experiences (none/1 or more, denotes as $death$), old-age $pension$ eligibility (whether the household has an aged 60-plus adult), numbers of non-residents (denoted as $migrants$, 0-15+), and household $size$ (0-20+). At the regional-level, we adjusted for an area's baseline Gini coefficient.

Overall, households have on average 7-8 people and with many circular migrants. About 44% of the households are headed by a female, a potential indication of having an absent male due to polygamy practice, labour migration, or AIDS-related death. Approximately 30% of households have at least one adult aged 60 or above, making them eligible to access the monthly mean-tested non-contributory public-sector old-age grant. The old-age grant remains

a primary source of stable income for most households. Supplementary Table 4 presents these descriptive results.

[Supplementary Table 4]

At the regional-level, the overall level of wealth inequality remained stable across the observed timeframe, from an average inequality score of 0.26 in 2016 to an average of 0.25 in 2018. Regions with a rising level of inequality appeared to be relatively wealthier at baseline (Supplementary Figure. 3). There are precisely 9 of these regions that have experienced an increase in the level of inequality ($\Delta\ Inq_v > 0$), with an increase ranging from about 0.6% to 6% (Supplementary Figure. 2, Panel c). However, considering that the analytical population is about 100-less in 2018 than in 2016 (Supplementary Table 3), it may be possible that the increase in inequality score is driven in part by changes in population size, particularly for smaller regions. We therefore restricted the regional-level binary outcome to areas with an increase in inequality score distributed at the upper 75th percentile ($\mu + (0.675)\sigma$), resulting in 6 of these regions with a rise in inequality score over time ($\Delta\ Gini_v \geq .02$).

[Supplementary Figure 3]

Supplementary Note 3. Analytical strategy

Analytical strategy

We took a stage-like approach to ensure our multilevel network models were not under- or over-parametrized. First, we examined the association between nodal attributes and economic outcomes both at the household- and regional-level, without accounting for network parameters. We ran a logistic regression predicting the likelihood for a household h to experience an outcome change ($\mathbf{1}_{\{\Delta\, w_h \neq 0\}}$ where $\Delta\, w_h = w_{h,2018} - w_{h,2016}$) by their baseline attributes described in Supplementary Note 2. We also ran a logistic regression predicting the likelihood for a community v to experience an outcome change ($\mathbf{1}_{\{\Delta\, Inq_v > 0\}}$ where $\Delta\, Inq_v = Inq_{v,2018} - Inq_{v,2016}$) by their baseline inequality score ($Inq_{v,2016}$). These models are formally defined in Equation 4 and 5, respectively.

$$\log(\frac{p(|\Delta\, w_h|>0)}{1-p(|\Delta\, w_h|>0)}) = \alpha + \beta_1 head + \beta_2 assets + \beta_3 death + \beta_4 pension + \beta_5 migrants + \beta_6 size + \varepsilon \quad (4)$$

$$\log(\frac{p(\Delta\, Inq_v))}{1-p(\Delta\, Inq_v)}) = \alpha + \beta_1 Inq_{v,2016} + \varepsilon \quad (5)$$

Supplementary Table 5 presents results from the logistic regressions with adjusted odds ratio and 95% confidence interval. Household-level results are discussed and visually displayed in the main text (Figure 2). At the regional-level, areas with a higher baseline inequality score were significantly less likely to experience a rise in their inequality index (OR = 0.3342; 95% CI [0.1170-0.9545]). We retained all attributes for the network model.

[Supplementary Table 5]

We next extended the current advances of network methodology (i.e., Autologistic Actor Attribute Models) to account for the multilevel, social-geographical dependency

structures illustrated in Figures 4 and 7 in the main text. The model is formally defined in Equation 1 in the main text.

Model fit and robustness

Goodness of fit

There are a large number of possible network configurations in the multilevel ALAAM specification. For configurations that involved network ties (A, B, X), we mainly focused on network configurations related to our theoretical interests illustrated in Figure 4 to ensure our final models were not overparametrized. However, given various possible combinations of $\{Y, A, B, X, Y^C\}$ in z_i, there may be other graph statistics that represent features of the observed, population-level data. We thus assessed the goodness of fit (GOF) statistics by simulating all implemented graph statistics in the MPNet software for the converged model (Wang et al. 2016; Koskinen and Snijders 2012). This modelling procedure is equivalent to that in Exponential Random Graph Models (ERGMs), which compares observed statistics with simulated samples from the converged model using t-ratios, with a value smaller than 0.1 indicating model convergence and an adequate representation to the observed data (Wang et al. 2016; Daraganova and Robins 2012; Koskinen and Snijders 2012). Specifically, we tested 99 types of graph statistics implemented in MPNet, based on a simulated sample of 3,000 outcomes vectors collected from 1 million Markov Chain Monte Carlo (MCMC) iterations.

The t-ratio from the GOF test for all parameters are smaller than 0.1 for the converged models, whereas a majority of non-fitted statistics are smaller than 2.0. These results suggest that our selected parameters provided an adequate fit to the observed data. However, there is a non-fitted network statistics that have a t-ratio greater than 2.0: A selection-based network contagion effect among the poorest households. We subsequently added the parameter and reran the GOF test.

The result suggests that network effects may be sorted based on a household's initial economic position, specifically among the poorest households: For the poorest households, having a network partner with the same asset status is correlated with an upward movement for both households. Supplementary Table 7 presents results from this estimate.

Robustness

In addition to GOF tests, we alternated various model specifications to validate the robustness of our main findings. First, a change in the household-level outcome ($|\Delta\, w_h| > 0$) can either indicates a downward movement ($\Delta\ w_{h,\ 2018<2016}$, assets lost), an upward movement ($\Delta\ w_{h,\ 2018>2016}$, assets accumulation), or asset stability over time ($\Delta\ w_{h,\ 2018=2016}$, stable asset status). To better understand these mobility patterns, we varied the outcome variable and ran additional ALAAMs predicting the likelihood of a household moving downward, upward, or remaining stable in their asset wealth status over the observed timeframe. Results from these models suggest that households are about 1.4 times ($e^{0.3390}$) significantly more likely to move downward if the same change occurred in their co-located network partners (Supplementary Table 8). Having social ties to those who are outside of the DSA is associated with an upward movement (Supplementary Table 9); and that socially and economically contagious upward movements are about 1.19 times ($e^{0.1730}$) significantly more likely to be observed in regions with an increase inequality score (Supplementary Table 9). Furthermore, households are about 0.87 times less likely to remain economically stable when connected to other households in the same *isigodi* ($e^{-0.1417}$), yet 1.37 times more likely to remain stable ($e^{0.3157}$) when their network partners also maintain stability (Supplementary Table 10).

A second concern derived from our analysis is that an increase in inequality score may be attributable to other contextual factors, such as changes in population structure in this highly mobile population. We thus evaluated the number of households in 2016 and 2018 for each

area and found that they remained relatively stable over the observed timeframe (Supplementary Table 3). However, our models cannot claim causality of these relationships (e.g., prior household- and regional-level economic conditions may also influence the organisation of social networks). While we are able to address some of the endogenous concerns by employing a multilevel ALAAM, and that our results are robust with alternative model specifications (Supplementary Table 11) – including to adjust for a range of observed attributes, network structural effects, the geographical locations of these networks, and dependencies in outcomes among networked partners (network autocorrelation) — it is important to note that we are unable to directly estimate the casual and effect between social networks and our outcomes, as well as to more precisely address other socio-cultural factors that may influence the strength of network effects (e.g., feeling more or less obligated to financially support (extended)families, see Mkhwanazi and Manderson 2020).

Lastly, we utilised a complete-case approach to our analyses, retaining observations without missing information on the household asset indicator in 2016 (n = 11,834) and 2018 (n = 11,734). These missing values can bias our model estimates, considering that the entire networked properties may be altered by "adding" a small amount of randomness to the network model (Watts and Strogatz 1998). Proposing a relevant method to address for missingness in social network data is beyond the scope of this work (for a detailed discussion, see Kossinets 2006). Nevertheless, we evaluate the missing data patterns derived from our key variable, household asset indicator (n = 1,160), to help us more precisely characterise the types of populations to whom our results most directly generalise (Supplementary Table 12). These results suggest that our analytic sample may underrepresent households with fewer members and migrants, as well as households without stable financial support from the old-age grant. These patterns suggest that being less socially connected may be related to non-response to the Household and Socio-Economic Survey (HSE) module.

Supplementary Tables

Supplementary Table 1. Multinomial logistic regression predicting the relative risk ratio (RRR) of having one (base), two, or three-plus household memberships among individuals in 2016, by age, sex, and marital status.

	Two household memberships					Three or more household memberships				
	**RRR**	**std. error**	***P* > |*t*|**	**95% CI**		**RRR**	**std. error**	***P* > |*t*|**	**95% CI**	
Sex										
Male (base)										
Female	0.4575	0.0925	0.0000	0.3078	0.6798	0.2204	0.2359	0.1580	0.0270	1.7962
Age	0.9251	0.0073	0.0000	0.9109	0.9396	0.9311	0.0253	0.0090	0.8827	0.9821
Sex * Age										
Female	1.0678	0.0114	0.0000	1.0457	1.0904	1.1495	0.0710	0.0240	1.0183	1.2974
Age^2	1.0009	0.0001	0.0000	1.0007	1.0010	1.0010	0.0003	0.0000	1.0005	1.0015
Sex * Age^2										
Female	0.9990	0.0001	0.0000	0.9988	0.9993	0.9975	0.0008	0.0030	0.9959	0.9992
Marital status										
Never-married (base)										
Married	0.9077	0.0773	0.2550	0.7682	1.0725	0.9382	0.3480	0.8640	0.4535	1.9412
Widowed	0.5851	0.0832	0.0000	0.4428	0.7731	0.7400	0.4570	0.6260	0.2206	2.4829
Separated/divorced	1.0704	0.4202	0.8630	0.4959	2.3105	3.2216	3.3729	0.2640	0.4139	25.0747
Informal union	1.1016	0.0554	0.0540	0.9983	1.2156	0.8079	0.1917	0.3690	0.5074	1.2864
Constant	0.2438	0.0351	0.0000	0.1839	0.3232	0.0087	0.0048	0.0000	0.0029	0.0256

Supplementary Table 2. Descriptive statistics for the one-mode inter-household social network and comparison with other network topologies.

Metric	Value	Notes
$\langle k \rangle$	1.7174	Average degree (mean number of connections per node), SD = 1.7966.
Cg	0.2077	Global clustering coefficient, indicating the overall likelihood that two neighbours of a node are connected.
$\langle C \rangle$	0.3032	Average local clustering coefficient, indicating the average probability that two neighbours of a node are connected. SD = 0.3769
$\langle l \rangle$	11.8418	Average path length, indicating the average number of steps along the shortest paths for all possible pairs of network node, SD = 2.9752
N	11,834	Total number of nodes in the network.
E	10,162	Total number of edges representing inter-household ties.
ω	4,068	Number of connected components in the network.
N_{max}	6,462	Number of nodes in the largest connected component of the network.

**Network Topologies**	$\langle C \rangle$	$\langle l \rangle$	$\langle k \rangle$
Observed	0.3032	11.8418	1.7174
Random	0.0000	15.5167	1.7255
Scale-Free	0.0000	10.9109	1.9998
Small-World	0.3783	13.0118	4.0000

Supplementary Table 3. The number of households by *izigodi* and study periods

**Administrative areas *(Izigodi)***	**2016 Freq.**	**%**	**2018 Freq.**	**%**
Isigodi 1	588	4.97	608	5.18
Isigodi 2	250	2.11	264	2.25
Isigodi 3	333	2.81	317	2.7
Isigodi 4*	1,926	16.28	1,891	16.1
Isigodi 5	663	5.6	587	5
Isigodi 6	184	1.55	194	1.65
Isigodi 7	643	5.43	637	5.42
Isigodi 8*	195	1.65	196	1.67
Isigodi 9*	107	0.9	109	0.93
Isigodi 10	24	0.2	24	0.2
Isigodi 11	505	4.27	500	4.26
Isigodi 12	320	2.7	322	2.74
Isigodi 13*	41	0.35	42	0.36
Isigodi 14	729	6.16	724	6.17
Isigodi 15	781	6.6	718	6.11
Isigodi 16	706	5.97	694	5.91
Isigodi 17*	1,343	11.35	1,372	11.68
Isigodi 18*	333	2.81	335	2.85
Isigodi 19	544	4.6	531	4.52
Isigodi 20	473	4	487	4.15
Isigodi 21	765	6.46	789	6.72
Isigodi 22	135	1.14	139	1.18
Isigodi 23	246	2.08	263	2.24
**Total**	11,834	100	11,743	100

To assess whether changes in both household-level and regional-level outcome are driven by changes in population demographics, we compared the number of households in 2016 and 2018 for each administrative area. The number of households remained relatively stable over time, though with about 100 fewer observed households in 2018. Regions that have experienced an increase in inequality score ($\Delta\ Inq_v > 0.2$) are highlighted with an asterisk sign (*).

Supplementary Table 4. Descriptive statistics on the distribution of baseline household- and regional-level attributes in 2016.

	N / Mean (SD)	**% / Median [IQR]**
**Household head sex**		
Male	6,624	55.97
Female	5,210	44.03
**Household asset status**		
Lowest	2,895	24.46
2nd lowest	2,418	20.43
Middle	2,321	19.61
2nd highest	2,148	18.15
Highest	2,052	17.34
**Mortality experiences**		
Zero-death	11,723	99.06
1/more death	111	0.94
**Pension-eligibility**		
No	8,225	69.5
Yes	3,609	30.5
**Household size**	7.901 (4.763)	7 [4-11]
**Non-residents**	4.052 (2.624)	4 [2-6]
**Baseline** Inq_v **score**	0.252 (0.039)	0.26 [0.228-0.277]
**Total** Households	11,834	100
Regions (*Izigodi*)	23	

Pension-eligibility is determined by whether the household has an adult aged 60-plus years old who qualifies for the monthly non-contributory old-age grant. Non-residents refer to adults aged 15-59 who are away for at least 6 months in a given year. These non-residents are typically considered as circular, labour-related migrants.

Supplementary Table 5. Logistic regressions predicting household-level ($\Delta\ w_{h,\,2018 \neq 2016}$) and regional-level ($\Delta\ Inq_{v,\,2018>2016}$) outcome changes, by baseline attributes.

	OR	std. error	$P > \|t\|$	95% CI	
Model I: Household-level (h)					
**Household head sex**					
Male (ref)					
Female	1.1200	0.0439	0.0040	1.0371	1.2096
**Household asset status**					
Lowest quintile (ref)					
2nd lowest	1.3179	0.0750	0.0000	1.1787	1.4735
Middle	1.1769	0.0675	0.0050	1.0517	1.3170
2nd highest	1.1624	0.0700	0.0120	1.0330	1.3081
Highest	0.4862	0.0308	0.0000	0.4295	0.5504
**Mortality experiences**					
Zero-death (ref)					
1/more death	0.7945	0.1563	0.2420	0.5404	1.1683
**Pension-eligibility**					
No (ref)					
Yes	1.2224	0.0519	0.0000	1.1247	1.3285
**N of non-residents**	1.2619	0.0340	0.0000	1.1971	1.3303
N of non-residents^2	0.9815	0.0019	0.0000	0.9778	0.9851
**Household size**	1.0351	0.0089	0.0000	1.0179	1.0526
Constant	0.6122	0.0371	0.0000	0.5437	0.6894
	**OR**	**std. error**	$P > \|t\|$	**95% CI**	
Model II: Regional-level (v)					
**Baseline Inq_v score (std.)**	0.3342	0.1789	0.0410	0.1170	0.9545
Constant	0.6122	0.0371	0.0000	0.5437	0.6894

Regional-level Gini scores at baseline are standardised (std). Robust standard errors applied for both models.

Supplementary Table 6. Multilevel ALAAM parameter estimates.

Effects	MPNet term	Parameters	std. error	*t*-ratio	*P* > |*t*|
**Baseline attributes** (households *h*)					
**Household head**	-				
Ref: Male head	-				
Female head	-	0.1183	0.0413	-0.0430	0.0042
**Household asset wealth**	-				
Ref: Lowest quintile	-				
2nd lowest quintile	-	0.2806	0.0583	-0.0338	0.0000
Middle quintile	-	0.1751	0.0589	0.0198	0.0029
2nd highest quintile	-	0.1609	0.0621	0.0018	0.0096
Highest quintile	-	-0.7110	0.0669	0.0353	0.0000
**Mortality experiences**	-				
Ref: no death	-				
Any death	-	-0.2645	0.2060	0.0493	0.1991
**Pension-eligibility**	-				
Ref: not eligible	-				
Eligible	-	0.2029	0.0443	-0.0288	0.0000
**Household size (std.)**	-	0.1595	0.0420	0.0403	0.0001
**Number of non-residents**	-	0.2304	0.0290	0.0008	0.0000
Number of non-residents^2	-	-0.0185	0.0021	0.0105	0.0000
**Ties strength**	-	0.0116	0.0050	0.0150	0.0213
**Baseline attribute** (*region v*)					
**Baseline *Inq*$_v$ score (std.)**	-	-1.3606	0.8504	0.0351	0.1096
**Household-level connectivity**					
Akin to constant	DensityA	-0.2675	0.0922	-0.0137	0.0037
Ego connectivity	Ego-2StarA	-0.0104	0.0076	-0.0122	0.1716
Closure	TA1A	0.0252	0.0284	0.0151	0.3756
**Regional-level connectivity**					
Akin to constant	DensityXB	-6.2906	3.6805	0.0291	0.0874
Akin to population size	EgoX-2StarB	0.0000	0.0000	-0.0122	0.3068
**Cross-level connectivity**					
Co-location (*h*)	TXAX-1A	-0.1354	0.0549	0.0339	0.0136
Cross-area (*h*)	L3XAX-1A	0.0094	0.0436	0.0060	0.8293
Co-location (*v*)	TXAX-B	-0.0362	0.0333	0.0149	0.2777
Cross-area (*v*)	L3XAX-1B	0.0007	0.0096	0.0206	0.9384
**Network interaction effects**					
Co-location (*h*)	TXAX-2A	0.2394	0.0838	0.0157	0.0043
Cross-area (*h*)	L3XAX-2A	-0.0230	0.0560	-0.0020	0.6808
Cross-area (*v*)	L3XAX-2B	0.0012	0.0116	0.0258	0.9206
**Cross-level interaction**	ContagionX-AB	0.0185	0.0275	0.0117	0.5005
**Micro-macro link**	AllTXAX	0.1024	0.0456	0.0083	0.0247

Parameter estimates are derived from the multilevel ALAAM and present as conditional log odds. The model included all baseline attributed in 2016 along with network parameters that are visually displayed in Figure 7 in the main text. Here, we retained the name of network parameters shown in the MPNet software. We note that the model also adjusted for a tie strength variable capturing the (mean) years in which households shared a tie through overlapping household members (0-16 years). The main, highlighted network parameters are visually displayed in Figure 5 in the main text.

Supplementary Table 7. Multilevel ALAAM parameter estimates with select-based network effects.

Effects	MPNet term	Parameters	std. error	*t*-ratio	*P* > |*t*|
**Baseline attributes** (households *h*)					
**Household head**	-				
Ref: Male head	-				
Female head	-	0.1140	0.0409	-0.0023	0.0054
**Household asset wealth**	-				
Ref: Lowest quintile	-				
2nd lowest quintile	-	0.3169	0.0637	0.0460	0.0000
Middle quintile	-	0.2098	0.0658	0.0100	0.0014
2nd highest quintile	-	0.1888	0.0681	0.0746	0.0055
Highest quintile	-	-0.4298	0.0901	-0.0150	0.0000
**Mortality experiences**	-				
Ref: no death	-				
Any death	-	-0.2785	0.2116	0.0273	0.1881
**Pension-eligibility**	-				
Ref: not eligible	-				
Eligible	-	0.2010	0.0477	0.0817	0.0000
**Household size (std.)**	-	0.1668	0.0395	0.0732	0.0000
**Number of non-residents**	-	0.2173	0.0271	0.0709	0.0000
Number of non-residents^2	-	-0.0174	0.0020	0.0860	0.0000
**Ties strength**	-	0.0125	0.0054	0.0257	0.0209
**Baseline attribute** (*region v*)					
**Baseline Inq_v score (std.)**	-	-1.2980	0.8082	0.0803	0.1083
**Household-level connectivity**					
Akin to constant	DensityA	-0.2991	0.0901	0.0342	0.0009
Ego connectivity	Ego-2StarA	-0.0044	0.0076	-0.0335	0.5615
Closure	TA1A	0.0084	0.0281	-0.0615	0.7645
**Regional-level connectivity**					
Akin to constant	DensityXB	-6.5311	3.7167	-0.0153	0.0789
Akin to population size	EgoX-2StarB	0.0000	0.0000	0.0085	0.2309
**Cross-level connectivity**					
Co-location (*h*)	TXAX-1A	-0.1131	0.0563	0.0285	0.0446
Cross-area (*h*)	L3XAX-1A	0.0379	0.0442	0.0129	0.3905
Co-location (*v*)	TXAX-B	-0.0325	0.0318	0.0049	0.3081
Cross-area (*v*)	L3XAX-1B	0.0020	0.0093	-0.0192	0.8296
**Network interaction effect**					
Co-location (*h*)	TXAX-2A	0.2127	0.0835	0.0202	0.0108
Cross-area (*h*)	L3XAX-2A	-0.0526	0.0585	0.0069	0.3684
Cross-area (*v*)	L3XAX-2B	-0.0009	0.0127	-0.0333	0.9418
**Cross-level interaction**	ContagionX-AB	0.0182	0.0234	-0.0056	0.4367
**Micro-macro link**	AllTXAX	0.0893	0.0444	0.0203	0.0444
**Selection-based network interaction**					
(Poorest households)	sesf1_oO-oOA	0.4729	0.1840	-0.0499	0.0102

Parameter estimates are derived from the multilevel ALAAM and present as conditional log odds. The highlighted parameter suggests that households in the poorest quintile are more likely to experience (upward) asset changes if same changes occurred among their network peers ("selection-based network interaction").

Supplementary Table 8. Multilevel ALAAM parameter estimates on downward asset changes ($\Delta\ w_{h,\ 2018<2016}$).

Effects	MPNet term	Parameters	std. error	*t*-ratio	*P* > |*t*|
**Baseline attributes (**households *h***)**					
**Household head**	-				
Ref: Male head	-				
Female head	-	0.3437	0.0474	0.0069	0.0000
**Household asset wealth**	-	0.7901	0.0199	0.0071	0.0000
**Mortality experiences**	-				
Ref: no death	-				
Any death	-	0.0875	0.2705	0.0858	0.7465
**Pension-eligibility**	-				
Ref: not eligible	-				
Eligible	-	-0.1984	0.0534	-0.0220	0.0000
**Household size (std.)**	-	0.0062	0.0504	0.0154	0.9027
**Number of non-residents**	-	-0.0595	0.0339	0.0188	0.0793
Number of non-residents^2	-	0.0001	0.0025	0.0167	0.0000
**Ties strength**	-	-0.0032	0.0062	-0.0005	0.6132
**Baseline attribute (***region v***)**					
**Baseline** Inq_v **score (std.)**	-	-2.7557	1.3683	0.0001	0.0440
**Household-level connectivity**					
Akin to constant	DensityA	-3.4501	0.1187	0.0145	0.0000
Ego connectivity	Ego-2StarA	-0.0144	0.0094	0.0039	0.1238
Closure	TA1A	0.0581	0.0348	-0.0127	0.0948
**Regional-level connectivity**					
Akin to constant	DensityXB	-7.9164	3.7798	0.0046	0.0362
Akin to population size	EgoX-2StarB	0.0000	0.0000	0.0500	0.1172
**Cross-level connectivity**					
Co-location (*h*)	TXAX-1A	-0.0549	0.0403	0.0573	0.1731
Cross-area (*h*)	L3XAX-1A	-0.0141	0.0364	0.0410	0.6989
Co-location (*v*)	TXAX-B	0.0388	0.0312	0.0335	0.2141
Cross-area (*v*)	L3XAX-1B	-0.0116	0.0099	0.0301	0.2431
**Network interaction effects**					
Co-location (*h*)	TXAX-2A	0.3390	0.1055	-0.0014	0.0013
Cross-area (*h*)	L3XAX-2A	-0.0543	0.0710	-0.0046	0.4440
Cross-area (*v*)	L3XAX-2B	0.0096	0.0091	0.0173	0.2938
**Cross-level interaction**	ContagionX-AB	0.0793	0.0421	0.0252	0.0596
**Micro-macro link**	AllTXAX	-0.0526	0.0905	0.0283	0.5610

Parameter estimates are derived from the multilevel ALAAM and present as conditional log odds. The model modified the outcome variable to assess the potentially economically downward movement given various network structural effects ($\Delta\ w_{h,\ 2018<2016}$, assets lost). We note that the baseline household asset indicator is introduced as a continuous variable, as treating it as an indicator variable led to model non-convergence (potentially due to empty cells as the poorest households cannot move downward in the economic ladder).

Supplementary Table 9. Multilevel ALAAM parameter estimates on upward asset changes ($\Delta\ w_{h,\ 2018>2016}$).

Effects	MPNet term	Parameters	std. error	*t*-ratio	*P* > |*t*|
**Baseline attributes (**households *h***)**					
**Household head**	-				
Ref: Male head	-				
Female head	-	-0.0740	0.0448	0.0424	0.0988
**Household asset wealth**	-	-0.8572	0.0198	-0.0269	0.0000
**Mortality experiences**	-				
Ref: no death	-				
Any death	-	0.3591	0.0491	-0.0379	0.0000
**Pension-eligibility**	-				
Ref: not eligible	-				
Eligible	-	-0.1984	0.0534	-0.0220	0.0000
**Household size (std.)**	-	0.1824	0.0441	-0.0807	0.0000
**Number of non-residents**	-	0.3575	0.0306	-0.0380	0.0000
Number of non-residents^2	-	-0.0250	0.0024	-0.0604	0.0000
**Ties strength**	-	0.0176	0.0061	-0.0184	0.0037
**Baseline attribute (***region v***)**					
**Baseline Inq_v score (std.)**	-	-2.7557	1.3683	0.0001	0.0440
**Household-level connectivity**					
Akin to constant	DensityA	0.6188	0.0969	0.0095	0.0000
Ego connectivity	Ego-2StarA	0.0030	0.0084	-0.0149	0.7251
Closure	TA1A	-0.0146	0.0285	-0.0439	0.6096
**Regional-level connectivity**					
Akin to constant	DensityXB	-4.1162	1.9837	-0.0445	0.0380
Akin to population size	EgoX-2StarB	0.0000	0.0000	-0.0682	0.6994
**Cross-level connectivity**					
Co-location (*h*)	TXAX-1A	-0.0627	0.0441	-0.0301	0.1544
Cross-area (*h*)	L3XAX-1A	0.0027	0.0389	0.0038	0.9446
Co-location (*v*)	TXAX-B	-0.0218	0.0215	-0.0482	0.3118
Cross-area (*v*)	L3XAX-1B	0.0115	0.0075	-0.0437	0.1267
**Network interaction effects**					
Co-location (*h*)	TXAX-2A	0.0253	0.0947	-0.0572	0.7889
Cross-area (*h*)	L3XAX-2A	0.0141	0.0620	-0.0182	0.8208
Cross-area (*v*)	L3XAX-2B	0.0030	0.0098	-0.0505	0.7581
**Cross-level interaction**	ContagionX-AB	-0.0575	0.0318	-0.0641	0.0708
**Micro-macro link**	AllTXAX	0.1730	0.0739	-0.0743	0.0191

Parameter estimates are derived from the multilevel ALAAM and present as conditional log odds. The model modified the outcome variable to assess the potentially economically upward movement given various network structural effects ($\Delta\ w_{h,\ 2018>2016}$, assets accumulation). We note that the baseline household asset indicator is introduced as a continuous variable, as treating it as an indicator variable led to model non-convergence (potentially due to empty cells as the wealthiest households cannot move upward in the economic ladder).

Supplementary Table 10. Multilevel ALAAM parameter estimates on stable asset status ($\Delta\ w_{h,\ 2018=2016}$).

Effects	MPNet term	Parameters	std. error	*t*-ratio	*P* > |*t*|
**Baseline attributes** (households *h*)					
**Household head**	-				
Ref: Male head	-				
Female head	-	-0.1183	0.0414	-0.0646	0.0043
**Household asset wealth**	-				
Ref: Lowest quintile	-				
2nd lowest quintile	-	-0.2812	0.0627	-0.0948	0.0000
Middle quintile	-	-0.1737	0.0557	0.0244	0.0018
2nd highest quintile	-	-0.1595	0.0610	-0.0522	0.0089
Highest quintile	-	0.7132	0.0663	-0.0023	0.0000
**Mortality experiences**	-				
Ref: no death	-				
Any death	-	0.2564	0.1983	0.0261	0.1960
**Pension-eligibility**	-				
Ref: not eligible	-				
Eligible	-	-0.2012	0.0455	0.0083	0.0000
**Household size (std.)**	-	-0.1604	0.0441	-0.0125	0.0003
**Number of non-residents**	-	0.0455	0.0287	-0.0579	0.1128
Number of non-residents^2	-	0.0184	0.0020	-0.0482	0.0000
**Ties strength**	-	-0.0115	0.0050	-0.0255	0.0217
**Baseline attribute** (*region v*)					
**Baseline Inq_v score (std.)**	-	-1.2674	0.8059	0.0232	0.1158
**Household-level connectivity**					
Akin to constant	DensityA	0.2733	0.0947	-0.0507	0.0039
Ego connectivity	Ego-2StarA	0.0104	0.0066	-0.0247	0.1185
Closure	TA1A	-0.0245	0.0263	-0.0463	0.3508
**Regional-level connectivity**					
Akin to constant	DensityXB	-4.0200	2.0901	0.0736	0.0544
Akin to population size	EgoX-2StarB	0.0000	0.0000	0.0266	0.7802
**Cross-level connectivity**					
Co-location (*h*)	TXAX-1A	-0.1417	0.0441	-0.0962	0.0013
Cross-area (*h*)	L3XAX-1A	0.0135	0.0361	-0.0482	0.7092
Co-location (*v*)	TXAX-B	0.0093	0.0247	0.0446	0.7074
Cross-area (*v*)	L3XAX-1B	0.0134	0.0095	0.0479	0.1592
**Network interaction effects**					
Co-location (*h*)	TXAX-2A	0.3157	0.0880	-0.0671	0.0003
Cross-area (*h*)	L3XAX-2A	-0.0224	0.0580	-0.0174	0.6998
Cross-area (*v*)	L3XAX-2B	0.0029	0.0104	0.0614	0.7844
**Cross-level interaction**	ContagionX-AB	-0.0468	0.0383	0.0483	0.2215
**Micro-macro link**	AllTXAX	-0.1141	0.0639	0.0437	0.0741

Parameter estimates are derived from the multilevel ALAAM and present as conditional log odds. The model modified the outcome variable to assess the stability of household asset wealth status given various network structural effects ($\Delta\ w_{h,\ 2018=2016}$, stable asset status).

Supplementary Table 11. Alternative model specifications with and without household- and regional-level controls

	MPNet term	Covariate free		Only Regional controls		Only Household-level controls		Main results	
		Parameters	std. error	Parameters	std. error	Parameters	std. error	Parameters	std. error
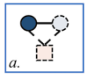	TXAX-1A	-0.1548	0.0543	-0.1570	0.0575	-0.1363	0.0570	-0.1354	0.0549
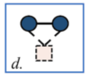	TXAX-2A	0.2492	0.0848	0.2584	0.0895	0.2358	0.0901	0.2394	0.0838
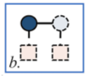	L3XAX-1A	0.0870	0.0411	0.0867	0.0397	0.0100	0.0464	0.0094	0.0436
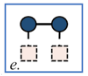	L3XAX-2A	-0.0200	0.0558	-0.0212	0.0562	-0.0242	0.0601	-0.0230	0.0560
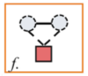	TXAX-B	-0.0456	0.0251	-0.0226	0.0296	-0.0513	0.0256	-0.0362	0.0333
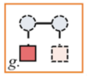	L3XAX-1B	0.0032	0.0068	-0.0046	0.0086	0.0047	0.0069	0.0007	0.0096
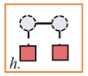	L3XAX-2B	0.0025	0.0085	0.0051	0.0092	0.0012	0.0085	0.0012	0.0116
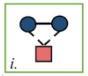	AllTXAX	0.1005	0.0420	0.0904	0.0425	0.1134	0.0425	0.1024	0.0456

Parameter estimates, derived from the multilevel ALAAMs, are presented as conditional log odds. Descriptions of the estimated parameters can be found in Fig. 4 of the main text. To benchmark these against our main results, we ran three additional models with and without various controls. Findings from these alternative specifications consistently align with our main results.

Supplementary Table 12. Missing data patterns on the outcome variable.

	N / %			
	**Observed**	**Missing**	**Total**	***Pearson's chi-square***
**Household head sex**				
Male	6,624	837	7,461	$p < 0.001$
	55.97%	72.16%	57.42%	
Female	5,210	323	5,533	
	44.03%	27.84%	42.58%	
**Mortality experiences**				
Zero-death	11,723	1,158	12,881	$p < 0.01$
	99.06%	99.83%	99.13%	
1/more death	111	2	113	
	0.94%	0.17%	0.87%	
**Pension-eligibility**				
No	7,269	998	8,267	$p < 0.001$
	61.42%	86.03%	63.62%	
Yes	4,565	162	4,727	
	38.58%	13.97%	36.38%	
				Bartlett's Test
**Temporary residents**				
Mean (SD.)	4.05 (2.62)	1.58 (1.35)		$p < 0.001$
Median [IQR]	4 [2-6]	1 [1-2]		
**Household size**				
Mean (SD.)	7.9 (4.76)	2.9 (2.57)		$p < 0.001$
Median [IQR]	7 [4.7]	2 [1-4]		

To characterise the types of populations to whom our results most directly generalise, we evaluate the missing data patterns derived from our key variable, household asset indicator (n = 1,160), by all observed indicators at baseline. These results show that our analytic sample may underrepresent households with fewer members and migrants (non-residents), as well as households without the access to the old-age grant.

Supplementary Figures

Supplementary Figure 1. Demographics of those with concurrent household memberships in 2016

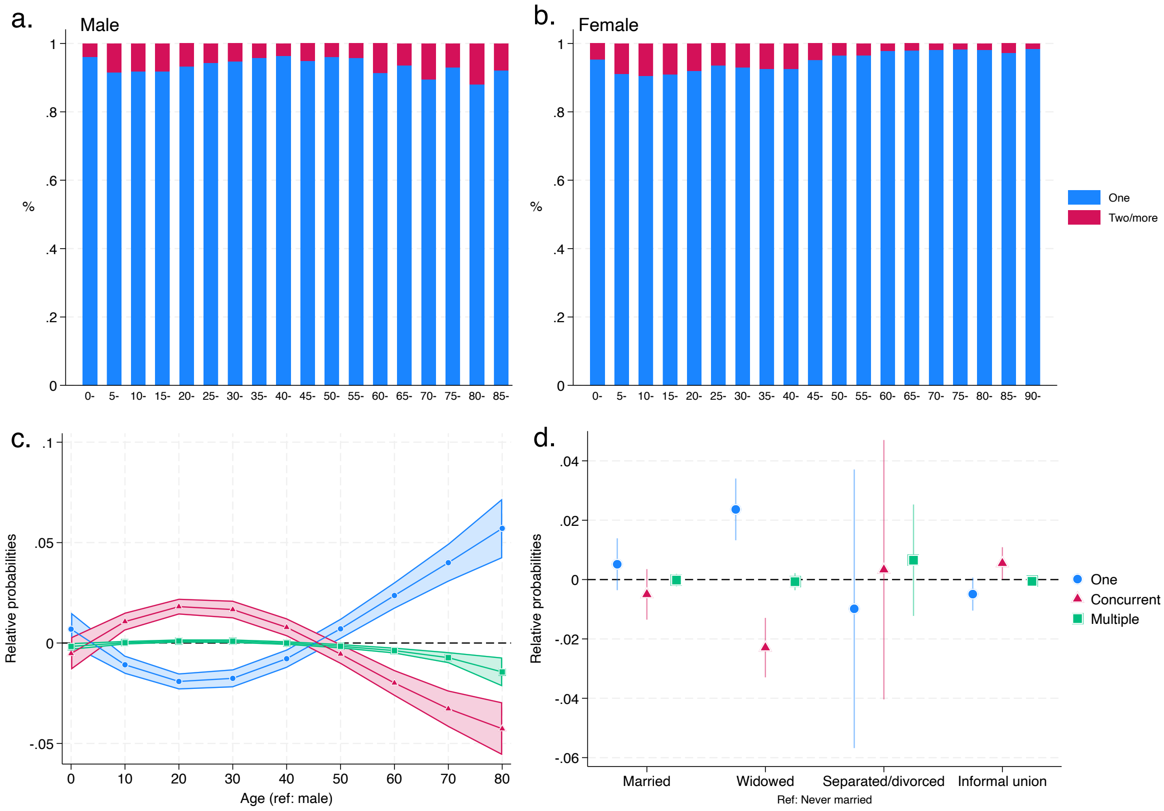

Panels **(a)** and **(b)** show the overall percentage of male and female with multiple concurrent household memberships by 5-years age groups, respectively. Descriptively, the prevalence of having multiple household memberships increases with age for male **(a)** while it decreases for female **(b)**. Panel **(c)** shows the average marginal estimates on the relative probability of having one, two, or multiple memberships for female (compared to male), by age. Finally, panel **(d)** shows the average marginal estimates on the relative probability of having multiple household memberships by marital status (compared to never-married). Estimates are derived from Equation 2.

Supplementary Figure 2. Comparing the degree distribution (K) of the observed network data with the network topologies of random, scale-free, and small-world network types.

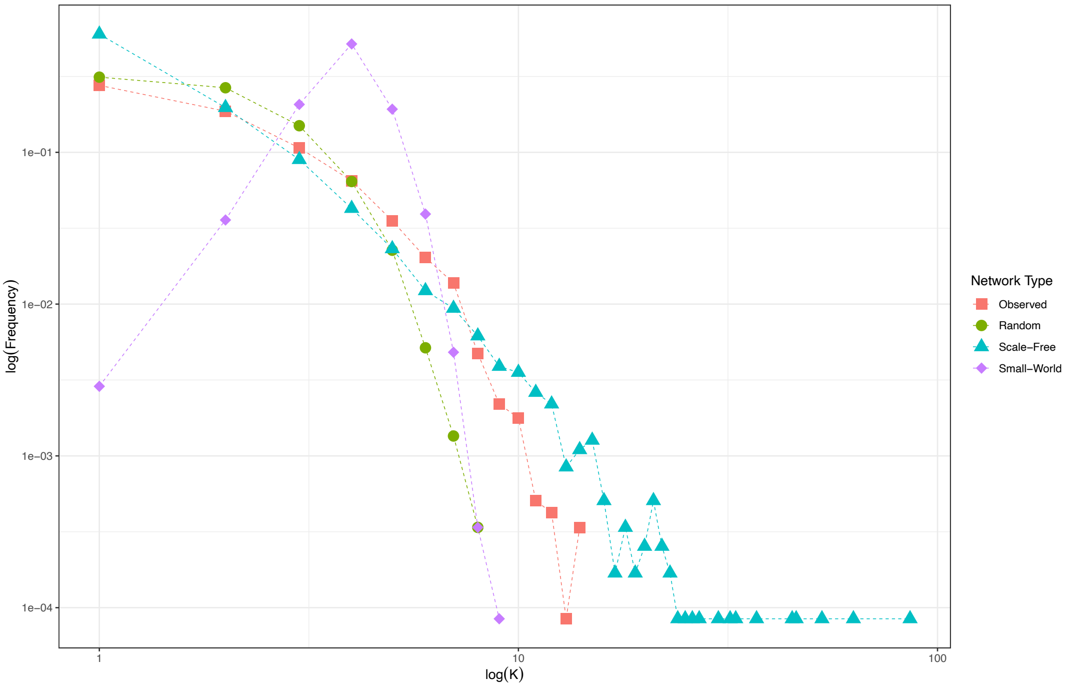

Supplementary Figure 3. Wealth distribution at baseline, by *izigodi*

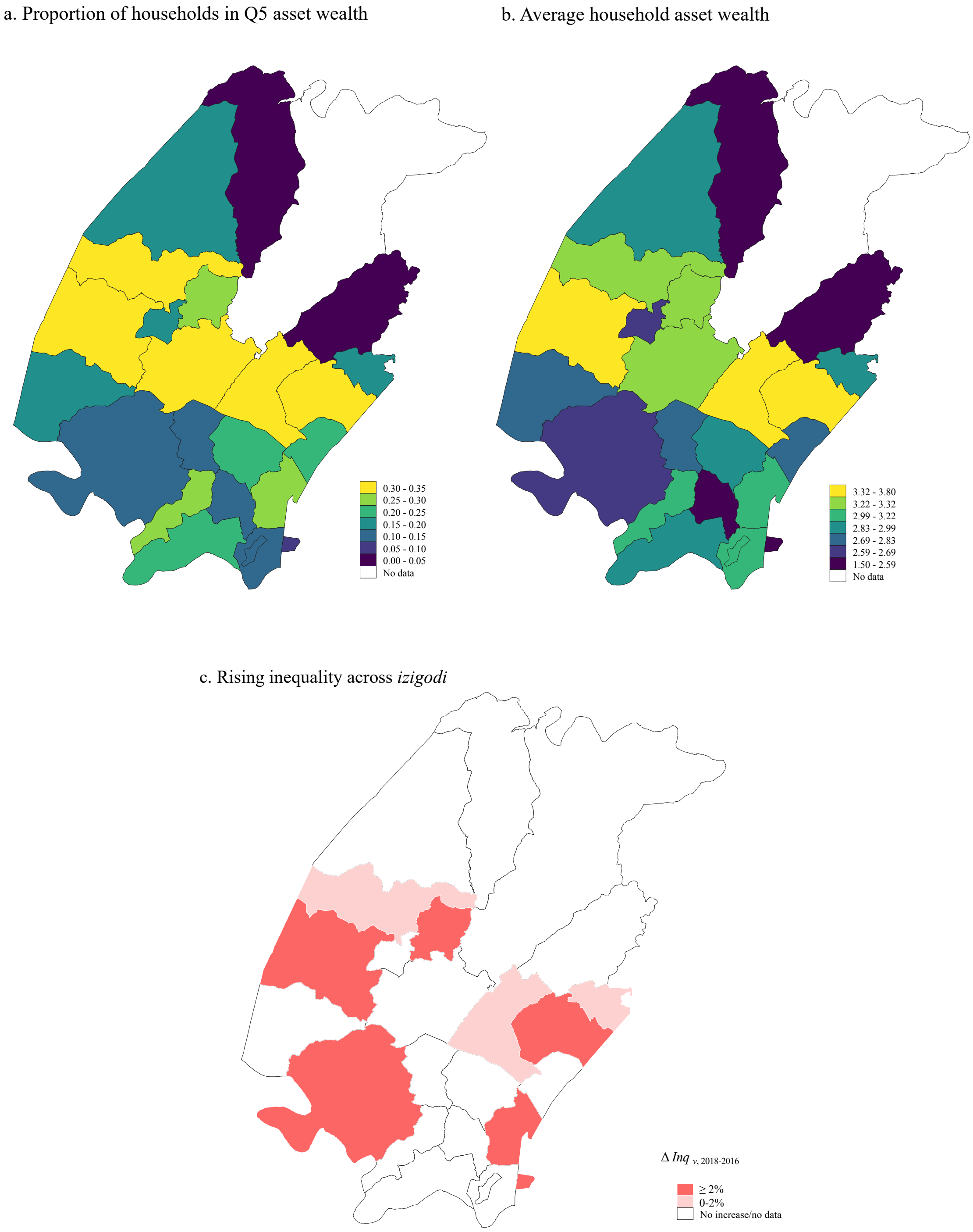

To understand the distribution of wealth across *izigodi* in 2016, we plot the proportion of households in the highest asset quintile (Q5) in panel **(a)** and the average household asset wealth status for each *isigodi* in panel **(b)**. Panel **(c)** displays the percentage increase in regional-level *v* inequality score over time ($\Delta\, Inq_v > 0$).

References

Barabasi, A. L. & Albert, R. Emergence of Scaling in Random Networks. *Science* **286**, 509-512 (1999)

Daraganova, G. & Robins, G. in *Exponential Random Graph Models for Social Networks: Theory, Methods, and Applications Structural Analysis in the Social Sciences* (eds Dean Lusher, Garry Robins, & Johan Koskinen) 102-114 (Cambridge University Press, 2012).

Erdös, P. & Rényi, A. On Random Graphs. I. *Publicationes Mathematicae (Debrecen)* **6**, 290-297 (1959).

Hosegood, V., Benzler, J. & Solarsh, G. C. Population mobility and household dynamics in rural South Africa: implications for demographic and health research. *Southern African Journal of Demography* **10**, 43-68 (2005).

Koskinen, J. & Snijders, T. in *Exponential Random Graph Models for Social Networks: Theory, Methods, and Applications Structural Analysis in the Social Sciences* (eds Dean Lusher, Garry Robins, & Johan Koskinen) 141-166 (Cambridge University Press, 2012).

Kossinets, G. Effects of missing data in social networks. *Social Networks* **28**, 247-268 (2006).

Mkhwanazi, N. & Manderson, L. *Connected Lives: Families, Households, Health and Care in South Africa*. Vol. 2020 (Ringgold Inc, 2020).

Spiegel, A., Watson, V. & Wilkinson, P. Domestic diversity and fluidity among some African households in Greater Cape Town. *Social Dynamics* **22**, 7-30 (1996).

Spiegel, A. The fludity of household composition in Matatiele, Transkei: a methodological problem. *African Studies* **45**, 17-35, doi: 10.1080/00020188608707648 (1986).

Russell, M. Understanding Black Households: The Problem. *Social Dynamics* **29**, 5-47 (2003).

Wang, P., Robins, G., Pattison, P. & Lazega, E. Social selection models for multilevel networks. *Social Networks* **44**, 346-362 (2016).

Wang, P., Robins, G., Pattison, P. & Koskinen, J. H. MPNet Program for the Simulation and Estimation of (p*) Exponential Random Graph Models. (2014).

Watts, D. J. & Strogatz, S. H. Collective dynamics of ‘small-world’ networks. *Nature* **393** (1998).
